# Effect of Different Karyophilic Peptides on Physical Characteristics and In Vitro Transfection Efficiency of Chitosan-Plasmid Nanoparticles as Nonviral Gene Delivery Systems

**DOI:** 10.1007/s12033-024-01087-9

**Published:** 2024-02-24

**Authors:** María Eugenia Aranda-Barradas, Héctor Eduardo Coronado-Contreras, Yareli Lizbeth Aguilar-Castañeda, Karen Donají Olivo-Escalante, Francisco Rodolfo González-Díaz, Carlos Gerardo García-Tovar, Samuel Álvarez-Almazán, Susana Patricia Miranda-Castro, Alicia Del Real-López, Abraham Méndez-Albores

**Affiliations:** 1 Unidad de Posgrado L4 (Laboratorio de Biotecnología), Universidad Nacional Autónoma de México, Facultad de Estudios Superiores Cuautitlán Campus 1, Av. 1o. De Mayo S/N Sta. María las Torres, 54740 Cuautitlán Izcalli, México; 2 Unidad de Investigación Multidisciplinaria L4 (Morfología Veterinaria y Biología Celular), Universidad Nacional Autónoma de México, Facultad de Estudios Superiores Cuautitlán Campus 4, Carretera Cuautitlán-Teoloyucan, Km 2.5 San Sebastián Xhala, 54714 Cuautitlán Izcalli, México; 3 Centro de Física Aplicada y Tecnología Avanzada, Universidad Nacional Autónoma de México, Blvd. Juriquilla 3001, Juriquilla La Mesa, 76230 Santiago de Querétaro, México; 4 Unidad de Investigación Multidisciplinaria L14-A1 (Ciencia y Tecnología de Materiales), Universidad Nacional Autónoma de México, Facultad de Estudios Superiores Cuautitlán Campus 4, Carretera Cuautitlán-Teoloyucan, Km 2.5 San Sebastián Xhala, 54714 Cuautitlán Izcalli, México

**Keywords:** Chitosan, Nanoparticles, Karyophilic peptides, Nuclear localization signal, Gene therapy

## Abstract

A strategy to increase the transfection efficiency of chitosan-based nanoparticles for gene therapy is by adding nuclear localization signals through karyophilic peptides. Here, the effect of the length and sequence of these peptides and their interaction with different plasmids on the physical characteristics and biological functionality of nanoparticles is reported. The karyophilic peptides (P1 or P2) were used to assemble nanoparticles by complex coacervation with pEGFP-N1, pQBI25 or pSelect-Zeo-HSV1-tk plasmids, and chitosan. Size, polydispersity index, zeta potential, and morphology, as well as in vitro nucleus internalization and transfection capability of nanoparticles were determined. The P2 nanoparticles resulted smaller compared to the ones without peptides or P1 for the three plasmids. In general, the addition of either P1 or P2 did not have a significant impact on the polydispersity index and the zeta potential. P1 and P2 nanoparticles were localized in the nucleus after 30 min of exposure to HeLa cells. Nevertheless, the presence of P2 in pEGFP-N1 and pQBI25 nanoparticles raised their capability to transfect and express the green fluorescent protein. Thus, karyophilic peptides are an efficient tool for the optimization of nonviral vectors for gene delivery; however, the sequence and length of peptides have an impact on characteristics and functionality of nanoparticles.

## Introduction

The approval of gene therapy-based treatments for several diseases by the Food and Drug Administration (FDA) and other regulatory agencies has been increasing in the last years [1]. Despite most of these treatments are based on viral vectors due to their high transfection efficiency, the potential immune response and the probability of insertional oncogenesis remain latent in this kind of vectors [2]. Hence, it is relevant to develop nonviral vectors that mimic the viral mechanisms of internalization and gene expression to enhance transfection efficiency without the disadvantages of viral vectors. Most of nonviral vectors are based on cationic molecules such as lipids, peptides, or polymers with certain characteristics, namely (1) the ability to protect the genetic material from the enzymatic degradation; (2) providing an extended blood circulation time; (3) directing the genetic material to a specific tissue or cell type; (4) degradation without the release of toxic subproducts; and (5) conferring the ability to cross the different physical and biological barriers [3]. Cationic polymers, widely used due to their content of amino functional groups, can be protonated at physiological or slightly acidic pH to form complexes with plasmids (pDNA) through electrostatic interactions, resulting in polyplex nanoparticles, which can be modified with biomolecules to direct polyplexes to a specific cell type or tissue. One of the most used cationic polymers is chitosan (CS), because of its low immunogenicity, high biocompatibility, and lack of intrinsic and/or subproducts toxicity. CS is a copolymer resulting from the deacetylation of chitin and is composed of aleatory distributed subunits of β-d-glucosamine and *N*-acetyl-d-glucosamine [4].

The in vitro transfection efficiency of CS nanoparticles (NPs) highly depends on CS deacetylation degree (DD), molecular weight (MW), pH, amine/phosphate (N/P) ratio, and other external factors, like the cell line physiology or the transfection protocol. Previously, we reported that using the complex coacervation method with low MW CS, high DD, and a N/P ratio of 8 (pEGFP-N1 plasmid), spherical NPs were obtained. Their size range was 150–200 nm with a slightly positive zeta potential (*ζ*-potential), demonstrating their biological functionality on cervical tumor cell lines [5].

Nevertheless, one of the reasons for the low transfection efficiency of nonviral vectors is the complicated nuclear internalization mechanism of therapeutic pDNA. In the classical pathway of nucleus entrance, α and β importins recognize cytoplasmic elements with a nuclear localization signal (NLS) and form complexes that interact with the RanGap1 enzyme, delivering the complexes to the nucleus by passing through the nucleoporins [6–8]. NLSs vary between species and proteins, but typically, these signals consist of one or more short sequences of positively charged lysine or arginine amino acids [8]. Thus, nucleic acids are not recognized by importins and only a few molecules will passively get into the nucleus. It has been reported that coupling NLSs through karyophilic peptides to pDNA before complex coacervation with CS or other polymers increases transfection efficiency [9–12]. However, although the addition of karyophilic peptides takes advantage of the nuclear import machinery, the incorporation method of the NLS peptide, its sequence and length, and its interactions and molar relation with pDNA need to be considered in the development of nonviral vectors for successful gene delivery [13].

In this research, two different karyophilic peptides CGGGPKKKRKVED (peptide 1 [P1], from SV-40 large T antigen) and PAAKRVKLD (peptide 2 [P2], from c-Myc) were coupled by electrostatic interactions to three plasmids with different sizes, pEGFP-N1, pQBI25 (both coding the green fluorescent protein), and pSELECT-zeo-HSV1-tk (coding *Herpes simplex* virus 1 thymidine kinase). Then, low MW CS was added to these pDNA-peptide complexes to obtain NPs by the complex coacervation method. Finally, it determined the effect of NLS sequence, length, and its interaction with pDNA on size, polydispersity index (PdI), *ζ*-potential, morphology, nuclear entry, and transfection capability of NPs.

## Materials and Methods

### Preparation of CS

The 20.6-kDa CS (85–90% DD) was obtained from shrimp exoskeleton and the resultant material was characterized according to the methodology reported by Miranda [14]. CS was dissolved in 0.1% acetic acid (CAS number 64-19-7, Sigma-Aldrich, USA) to obtain a 1.0% CS stock solution.

### Propagation and Isolation of Plasmids

pEGFP-N1 (4733 bp) and pQBI25 (6238 bp) were kindly donated by Mirna Olivia Martínez and Rubén Sánchez, respectively. pHSV1-tk (4274 bp) was purchased from Invivogen (USA). The three plasmids were propagated according to the following procedure: 500 mL of Luria–Bertani medium (Conda, Spain) were supplemented with 50-μg/mL antibiotic [pEGFP-N1: kanamycin (Sigma, USA), pQBI25:ampicillin (Sigma, USA), and pHSV1-tk:zeocin (Invivogen, USA)] before inoculation with transformed *Escherichia coli* and incubated at 37 °C during 16–18 h. Plasmid isolation was made using the Plasmid Mega kit (QIAGEN, USA) and was quantified with a spectrophotometer Epoch (Biotek, UK).

### NPs Preparation by the Complex Coacervation Methodology

**Fig. 1:**
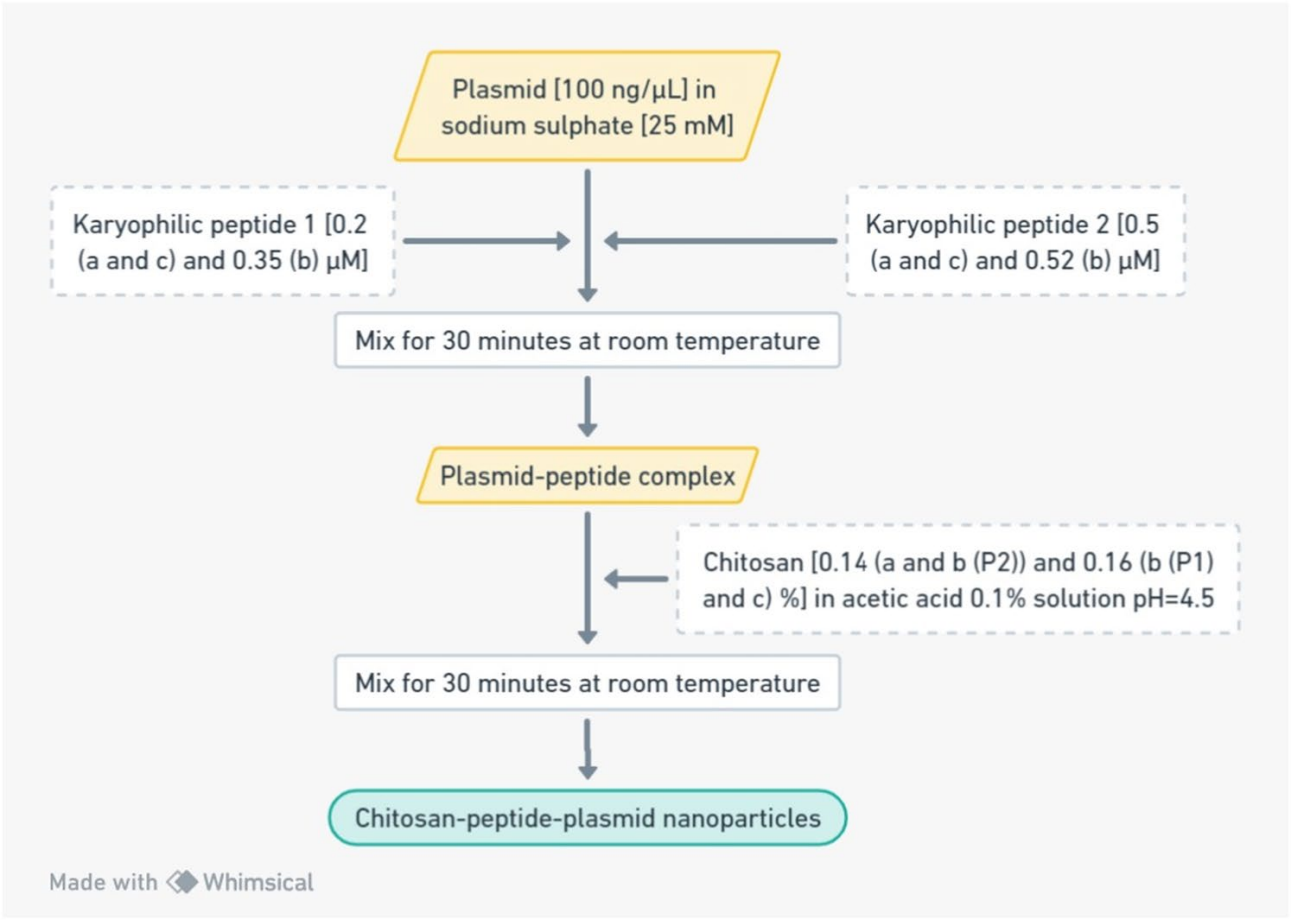
General procedure for the assembly of CS-based NPs using the complex coacervation method. (a) pEGFP-N1; (b) pQBI25; and (c) pHSV1-tk

The plasmid solutions were prepared at a constant concentration of 100 μg/mL using Na_2_SO_4_ (25 mM) as diluent. An equal volume of the karyophilic peptide (at a given concentration) was added to the pDNA and mixed for 30 min at room temperature. Then, one volume of CS solution was added, maintaining a relation 1:1:1 pDNA:karyophilic peptide:CS and then mixed again for 30 min (Fig. 1).

### pDNA-Karyophilic Peptides Shift Assays

To determine the optimal concentration of the peptides, stock solutions of karyophilic peptide 1 (P1: Large T-antigen NLS; sequence: CGGGPKKKRKVED) and 2 (P2: c-Myc NLS; sequence: PAAKRVKLD) were prepared to obtain the following concentrations: pEGFP-N1: P1: 0.03, 0.07, 0.15, 0.2, 0.3, and 0.5 μM and P2: 0.03, 0.07, 0.3, 0.5, 0.8, and 1.0 μM; pQBI25: P1: 0.25, 0.3, 0.35, 0.4, 0.45, and 0.5 µM and P2: 0.19, 0.27, 0.36, 0.44, 0.52, and 0.59 µM; and pHSV1-tk1: P1: 0.05, 0.1, 0.15, 0.2, 0.25, and 0.3 µM and P2: 0.2, 0.3, 0.4, 0.5, and 0.6 µM. Equal volumes of each peptide solution were added to 100-μg/mL pDNA (for each plasmid) and mixed for 30 min at room temperature. Samples were run in a 1% agarose gel at 100 mV for 30 min.

### pDNA-Karyophilic Peptides-CS Shift Assays

To determine the optimal concentration of CS to form NPs, the following concentrations of CS solutions were prepared from the stock (see “Preparation of CS” section) and the pH value was adjusted to 5.5 with 1% NaOH. pEGFP-N1: 0.03, 0.04, 0.6, 0.8, 0.1, 0.12, and 0.14% (w/v); pQBI25: 0.06, 0.08, 0.1, 0.12, 0.14, and 0.16% (w/v); and pHSV1-tk: 0.04, 0.06, 0.08, 0.10, 0.12, 0.14, and 0.16% (w/v). An equal volume of each solution was added to pDNA (100 μg/ml), which previously was complexed with P1 or P2 at the selected concentration on the pDNA-karyophilic peptides shift assays, maintaining a proportion 1:1:1 and mixing for 30 min at room temperature. Samples were run in a 1% agarose gel at 100 mV for 30 min.

### Scanning Electron Microscopy (SEM)

The size and morphology of the NPs were scrutinized using a JEOL JSM- 6060LV Scanning Electron Microscope (JEOL Inc., USA). The samples were coated with a thin gold layer to enhance electron conductivity and image quality. The microscopy analyses were performed with an accelerating voltage of 20 keV.

### Dynamic Light Scattering (DLS)

The NPs size was determined using DLS with the instrument ZetaSizer Pro (Malvern, UK). Samples were measured in a DTS1070 disposable cell by triplicate considering “chitosan” in the software material management and water viscosity. The size distribution and PdI were adjusted to correlation function and algorithms of the ZetaSizer software.

### Laser Doppler Electrophoretic Mobility

The *ζ*-potential of NPs was determined by Laser Doppler Electrophoretic Mobility with the ZetaSizer Pro instrument (Malvern, UK). The Smoluchowski function was selected at a 1.5 value in the instrument software.

### Biological Activity Evaluation

#### Cell Culture

The HeLa cell line (gently donated by Dr. Elizabeth Ortíz Sánchez) was cultured in Dulbecco’s Modified Eagle Medium (DMEM; ThermoFisher, USA) supplemented with 10% fetal bovine serum (FBS; ThermoFisher, USA) at 37 °C and 5% CO_2_ to obtain 150,000 cells/well in a 24-well plate with cover slides at the bottom of the wells. When a 50% of confluence was reached, the complexes were mixed with supplemented DMEM and added to the cultures (see “Internalization Assays” and “Transfection Assays” sections).

#### Internalization Assays

Before the complex formation, the plasmid was stained with propidium iodide and NPs were assembled as previously described. These complexes were added to HeLa cell cultures (with cover slides on the bottom of the wells) and incubated for 15, 30, and 60 min at 37 °C and 5% CO_2_. After these incubation times, NPs were retired and a water solution of 4% paraformaldehyde was added to fix the samples. Then, the cover slides were transferred to a glass slide and the mounting medium with 4′, 6-diamidino-2-phenylindole (DAPI; Abcam, USA) was used. Samples were stuck to glass slides using nail polish and were finally observed under the Axio scope 40 fluorescence microscope (Zeiss, Germany) at × 200 or × 400 magnifications.

#### Transfection Assays

The complexes were added to HeLa cells and incubated for 16 h at 37 °C and 5% CO_2_. Then, this NPs medium was replaced with a fresh supplemented medium and the culture was incubated for another 48 h. After incubation, the medium was retired, and 4% paraformaldehyde was added and incubated for 15 min at room temperature. Samples were washed three times with phosphate-buffered saline (PBS) solution. The cover slides were mounted using a mounting medium with DAPI (Abcam, USA) and stuck to glass slides using nail polish. Samples were finally observed under the Axio scope 40 fluorescence microscope using the DAPI, rhodamine, or FITC filter sets (Zeiss, Germany) at × 200 magnifications.

### Experimental Design and Statistical Analysis

The size, ζ-potential, and PdI of NPs were determined as a completely randomized design, with three replicates. Data were analyzed by the Student’s *t* Test and one-way ANOVA with Tukey’s Test using the GraphPad Prism 9 software (Dotmatics, USA).

## Results

### Interaction of pDNA with the Karyophilic Peptides

**Fig. 2:**
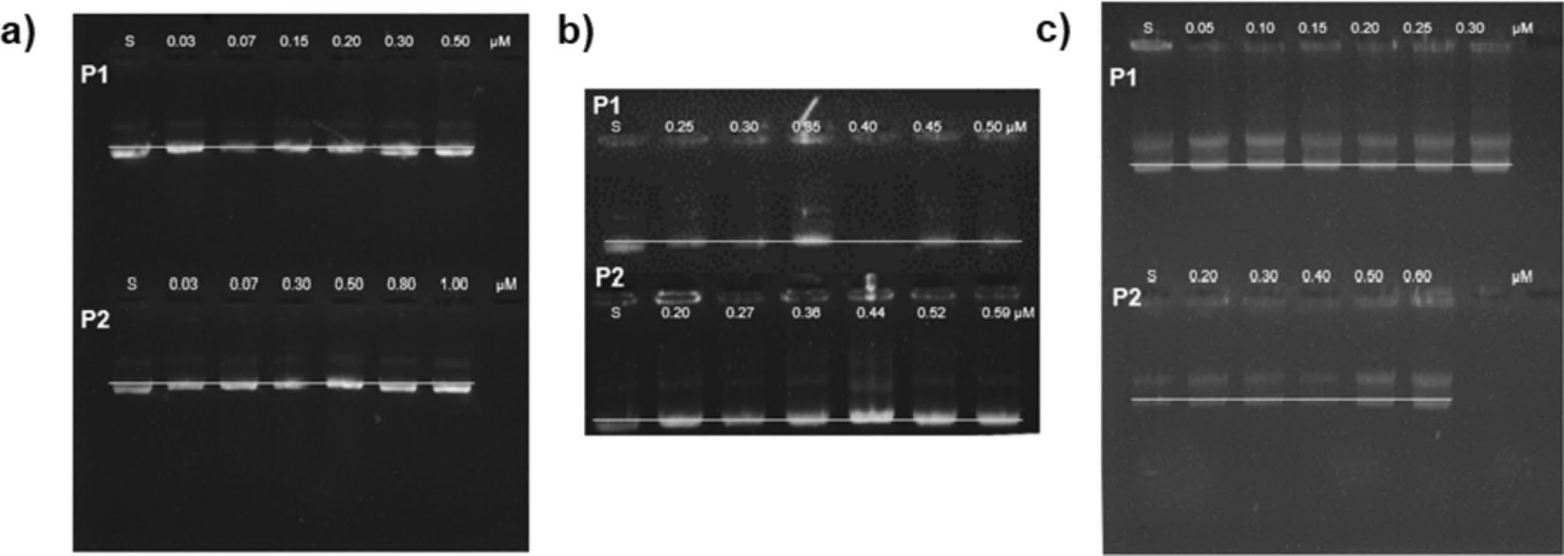
Electrophoretic shift assays for each plasmid with increasing concentrations of P1 and P2 in 1% agarose gel. **a** pEGFP-N1, **b** pQBI25, and **c** pHSV1-tk. *S* free plasmid sample

To determine the optimal ratios for assembling pDNA-karyophilic peptide complexes, electrophoretic shift assays were performed using increasing concentrations of the karyophilic peptides. In comparison to free pDNA, the migration of pDNA-karyophilic complexes was slightly delayed (Fig. 2). According to Hernández-Baltazar et al*.* [9], the migration band located in the middle of the plasmid control band represents the concentration at which the peptide interacts properly with the plasmid. However, this interaction does not saturate the negative charges of the pDNA, allowing it to further interact with CS. For P1 karyophilic peptide, optimal concentrations were 0.2, 0.35, and 0.2 μM, and for P2, the optimal concentration were 0.5, 0.52, and 0.5 μM for pEGFP-N1, pQBI25, and pSelect-Zeo-HSV1-tk, respectively (Fig. 2).

### Interaction of pDNA-Karyophilic Complexes with CS

**Fig. 3:**
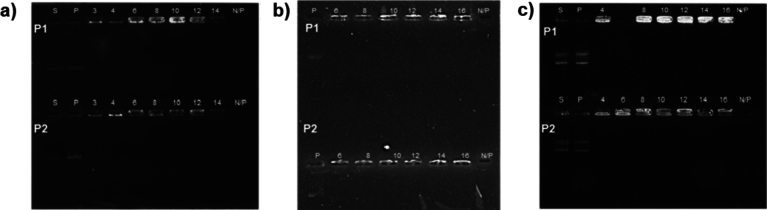
Electrophoretic shift assays in 1% agarose gel of pDNA-karyophilic peptide complexes and CS using different N/P ratios. **a** pEGFP-N1, **b** pQBI25, and **c** pSelect-Zeo-HSV1-tk. *S* free plasmid, *P* pDNA-karyophilic peptide complex, *N/P* N/P ratio (amine–phosphate ratio)

Once the optimal concentrations of the karyophilic peptides were determined, the N/P ratio was also assessed also through shift assays. NPs were assembled by adding increasing concentrations of CS to the previously obtained pDNA-karyophilic complexes. Based on our previous experience and the total compaction and retention of pDNA, the optimal N/P ratios were as follows: 14 for pEGFP-N1 for both peptides (Fig. 3a), for pQBI25, 16 and 14 for P1 and P2, respectively (Fig. 3b), and 16 for p-Select-Zeo-HSV1-tk for both peptides (Fig. 3c).

### Physical Characterization of NPs

**Fig. 4:**
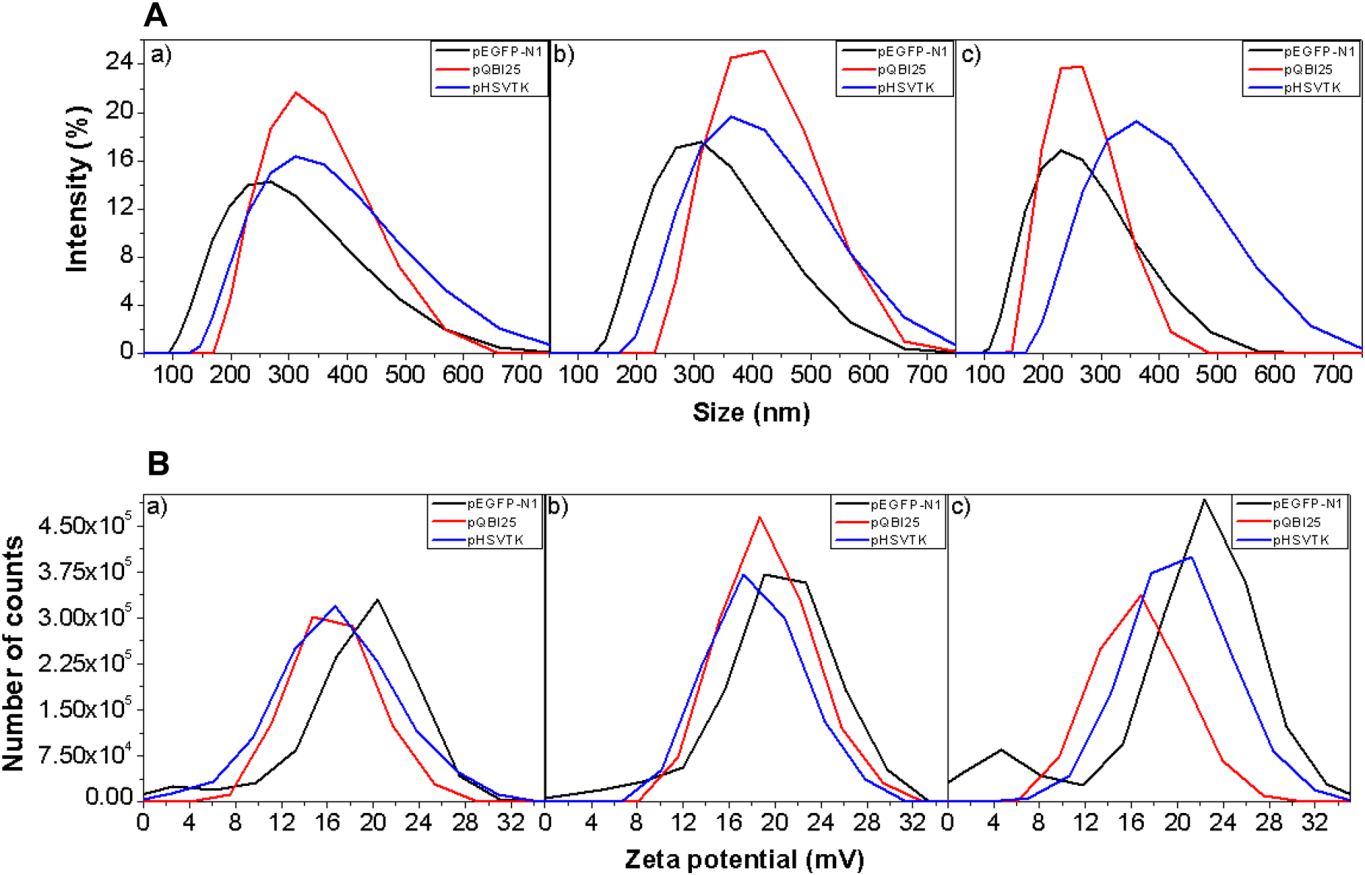
Representative graphics of **A** size and **B** ζ-potential of the NPs for pEGFP-N1 (black line), pQBI25 (red line), and pSelect-Zeo-HSV1-tk (blue line). (a) Control NPs (no peptide), (b) P1 NPs, and (c) P2 NPs

To determine the size and PdI of the NPs, complexes were analyzed by DLS (Fig. 4A) and *ζ*-potential was assessed by Laser Doppler velocimetry (Fig. 4B). In general, the size of control NPs (without peptides) was around 250 nm. The addition of P1 resulted in larger NPs, whereas the incorporation of P2 decreased the size of NPs. On the other hand, the control NPs had *ζ*-potential values of approximately 14 to 20 mV, which increased upon the incorporation of P1, with P2 leading to the highest *ζ*-potentials.

Table 1 summarizes the results for each NP according to the plasmid and karyophilic peptide used for their assembly.

**Table 1:** Results of size, PdI, and *ζ*-potential of NPs according to the plasmid and karyophilic peptides (*n* = 3)

Plasmid	Sample	Particle size (nm)	*ζ*-Potential (mV)	PdI
pEGFP-N1	Control	217.86 ± 18.02	19.64 ± 1.16	0.105 ± 0.04
	P1 NPs	299.97 ± 20.86	19.70 ± 1.38	0.213 ± 0.04
	P2 NPs	220.65 ± 18.80	20.76 ± 1.36	0.077 ± 0.02
pQBI25	Control	327.56 ± 29.21	16.5 ± 2.8	0.14 ± 0.07
	P1 NPs	346 ± 28.52	17.13 ± 4.27	0.15 ± 0.23
	P2 NPs	262.2 ± 0.6	16.56 ± 1.28	0.10 ± 0.12
pHSV1-tk	Control	353.43 ± 61.57	13.81 ± 3.70	0.32 ± 0.21
	P1 NPs	375.70 ± 19.10	15.84 ± 2.71	0.44 ± 0.53
	P2 NPs	321.13 ± 81.79	16.35 ± 2.57	0.45 ± 0.39

Student’s *t* Test and one-way ANOVA with Tukey’s Tests were conducted to determine the significant differences in the comparisons between characteristics of the NPs based on the plasmid and/or karyophilic peptide used for their assembly.

**Fig. 5:**
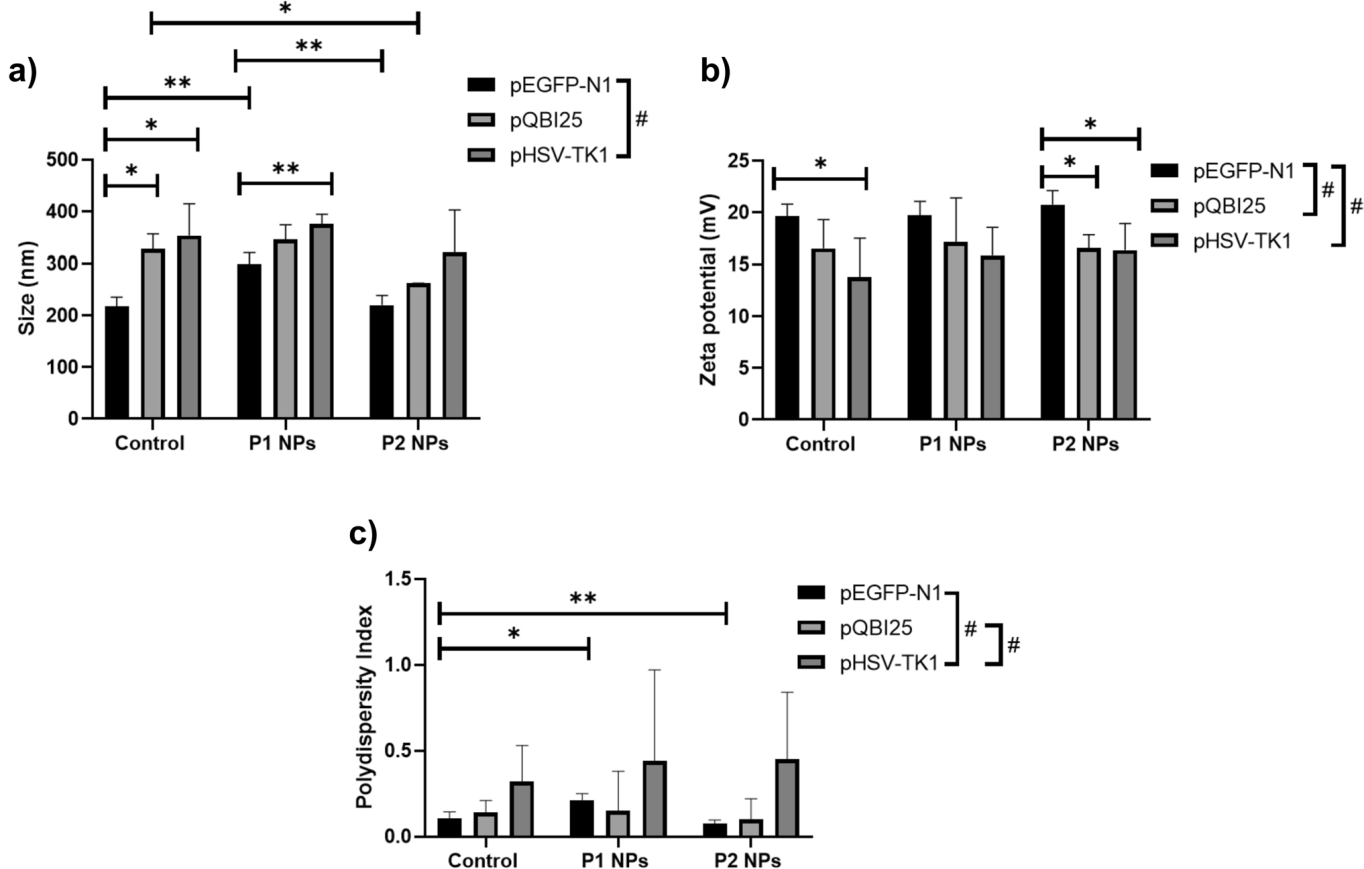
Summary of Student’s *t* Tests between each mean to stablish the comparison of physical characteristics of NPs based on the presence or absence of karyophilic peptides (P1 or P2). **a** Particle size, **b** *ζ*-potential, and **c** polydispersity Index (PdI). Data expressed as the mean ± SD; *p < 0.05; **p < 0.01 for Student’s *t* Tests. Additionally, the effect of the plasmid size was analyzed by one-way ANOVA and compared with Tukey’s Test. Significant differences were considered as ^#^*p* < 0.05; *n* = 3

Figure 5 illustrates the significance regarding size, *ζ*-potential, and PdI for the three plasmids comparing control NPs with P1 or P2 NPs and P1 with P2 NPs. Based on the significance of the comparisons, these results suggest that the addition of either P1 or P2 on pSelect-Zeo-HSV1-tk NPs has no impact on their size, PdI, and *ζ*-potential. For the other two plasmids, the P2 NPs showed a significantly smaller size compared to the control (pQBI25) or P1 (pEGFP-N1) NPs (Fig. 5a). According to *p* values, pEGFP-N1 NPs have the smallest particle size, PdI (except for P1 NPs) and the highest *ζ*-potential values compared to the other two plasmids NPs (Fig. 5a–c). The addition of P1 to pEGFP-N1 NPs significantly increased the PdI, while the addition of P2 to this plasmid NPs significantly decreased this parameter, suggesting that P2 resulted in a more homogeneous population of particles.

**Fig. 6:**
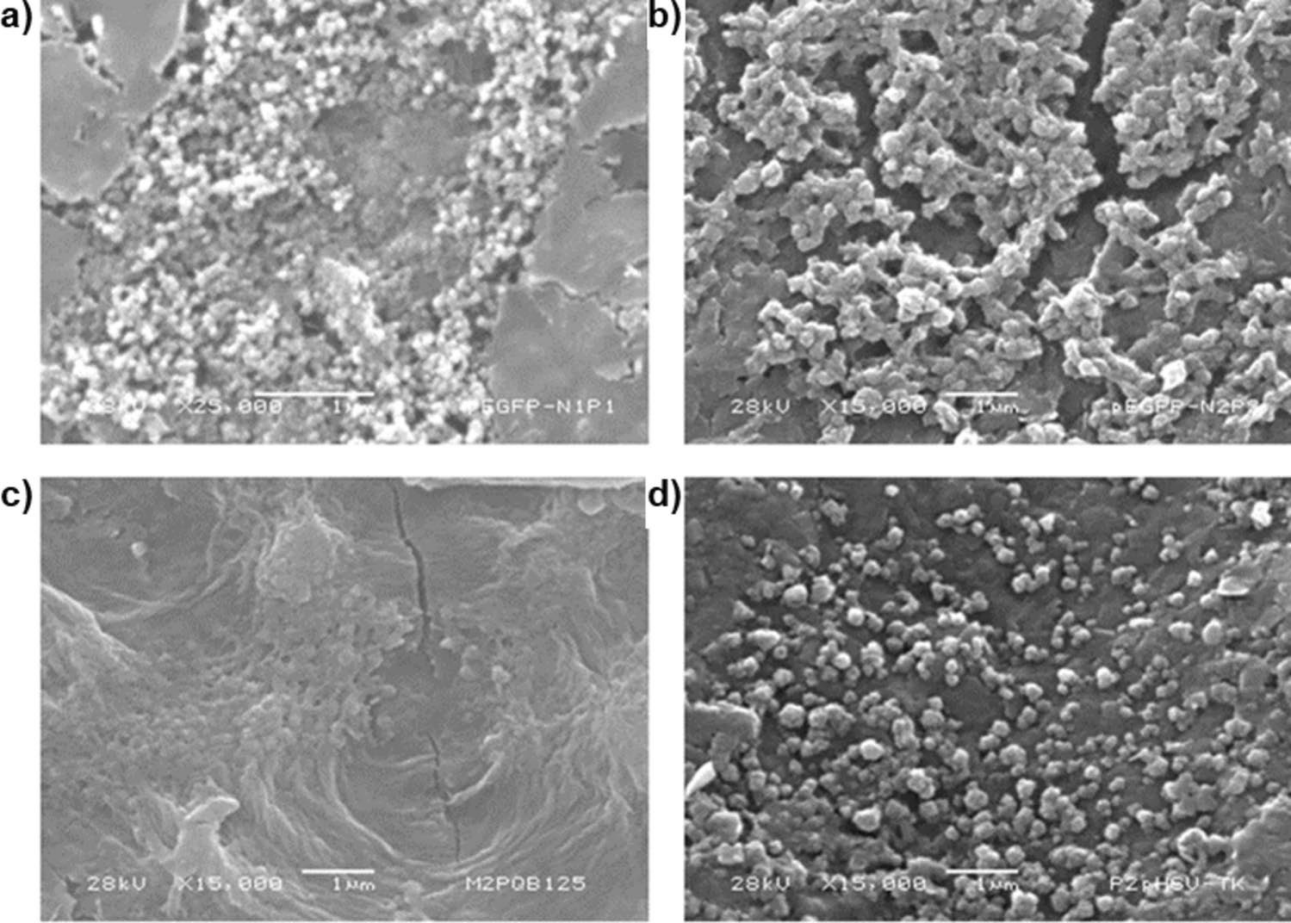
Morphology of NPs formulated with different plasmids. **a** pEGFP-N1 P1 NPs at × 25,000, **b** pEGFP-N1 P2 NPs at × 15,000, **c** pQBI25 P2 NPs at × 15,000, and **d** pHSV1-tk P2 NPs at × 15,000. *Scale bar* 1 µm

Due to the significant difference found between pEGFP-N1 P1 and P2 NPs, their morphology, assessed by SEM, was compared with the morphology corresponding to NPs with the best physical characteristics between P1 and P2 for the other two plasmids. Micrographs of pEGFP-N1 P1 (Fig. 6a) and P2 (Fig. 6b) NPs and pQBI25 P2 NPs (Fig. 6c) and pSelect-Zeo-HSV1-tk P2 NPs (Fig. 6d) show that all NPs have spherical forms and are comparable to the morphology of pEGFP-N1 control NPs previously reported by our research group [5].

### In Vitro NPs Internalization

**Fig. 7:**
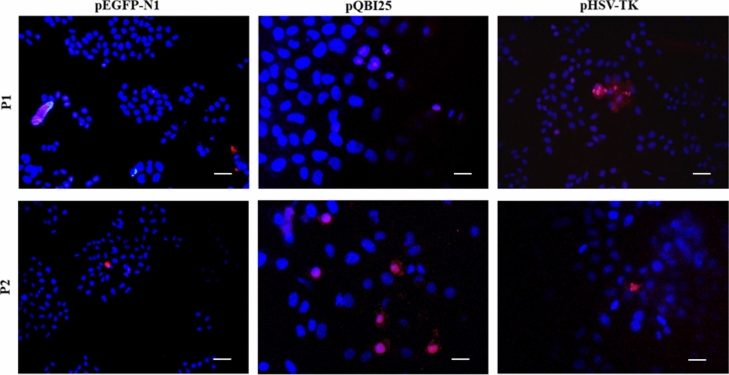
Internalization of NPs on HeLa cells after 30 min of addition. It showed the merge of DAPI (blue) and propidium iodide-labeled NPs plasmids (red). *Scale bar* pEGFP-N1 (P1 and P2) and pHSV1-tk P1: 10 µm (× 200 total magnification); pQBI25 (P1 and P2) and pHSV1-tk P2: 5 µm (× 400 total magnification)

Cell and nuclear internalization of NPs were assessed using propidium iodide-labeled plasmid to assemble the NPs. After 15 min of interaction between NPs with HeLa cells, this red label was observed on the nuclear periphery. After 30 min of exposure to NPs (Fig. 7), plasmids were already localized inside the nucleus when the karyophilic peptides were used. Control NPs did not show cell internalization even at 60 min, although entry after longer times is not ruled out and would explain previously reported results [5].

### Transfection Capability of NPs

**Fig. 8:**
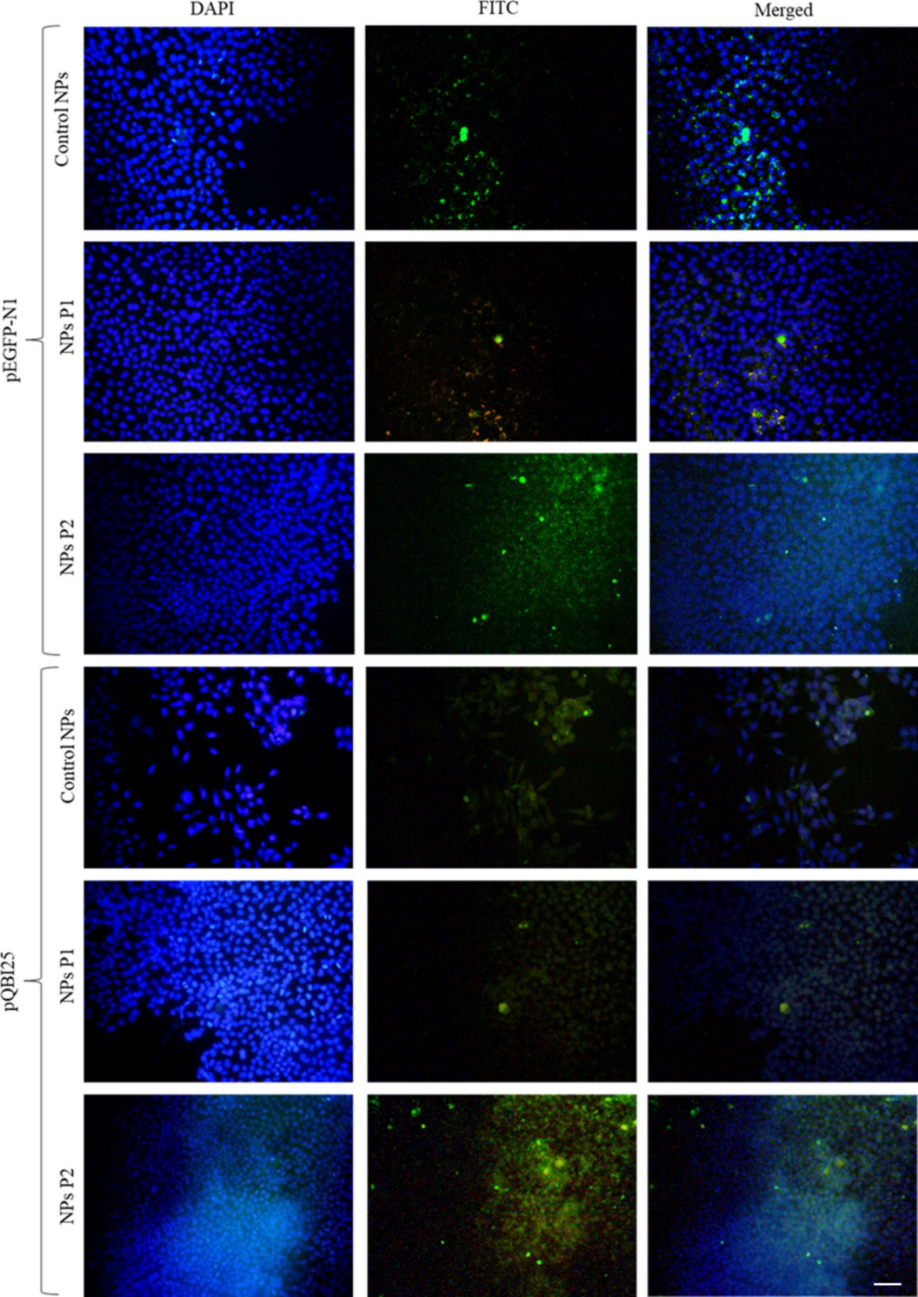
Green fluorescent protein expression on HeLa cells after transfection with pEGFP-N1 and pQBI25 control, P1, and P2 NPs, respectively. *Scale bar* 10 µm; × 200 total magnification

HeLa cells were transfected with pEGFP-N1 and pQBI25 control, P1, and P2 NPs. Figure 8 qualitatively shows that the presence of P2 increased the biological functionality of NPs and consequently, the expression of the green fluorescent protein (for both plasmids NPs), as compared with control and P1 NPs.

## Discussion

Electrophoretic shift assays demonstrated that CS interacts with and fully condense plasmids even after their interaction with the determined concentrations of the karyophilic peptides (Figs. 2, 3). This finding is consistent with the report of Hernández-Baltazar et al. (2012) for poly-l-lysine-based polyplexes [9]. Generally, karyophilic peptides by themselves can slightly condense DNA, as NLSs consist of at least three positively charged lysine molecules [8, 13, 15]. However, the peptide length and sequences including neutral amino acids could result in a lower rate of pDNA condensation, and this is reflected in the size of the NPs (Fig. 5a).

For pEGFP-N1 and pQBI25, P1 NPs showed a significantly larger size compared to control and P2 NPs. This suggests that P1 is not capable of compacting pDNA as efficiently as P2, and this cannot be compensated by further interaction with CS. The P1 contains more neutral amino acids, preventing electrostatic interaction with pDNA and hence cannot contribute to its condensation. This interference with the interaction with CS can, in turn, raise the particle size, despite the determination of optimal molar ratios by electrophoretic shift assays.

In contrast, the shorter length and lower content of neutral amino acids in P2 promote its electrostatic interaction with pDNA and between pDNA-peptide complex and CS. This leads to higher condensation and smaller NPs sizes, facilitating their internalization, consistent with in vitro experiments (Fig. 7). Manzanares and Ceña (2020) reported that most of the CS submicron NPs mainly enter the cells by clathrin-mediated endocytosis independently of their size [16].

Nevertheless, it has been reported that other NPs with sizes of 150–200 nm are mostly internalized via clathrin- or caveolin-mediated endocytosis, while NPs from 250 nm to 3 μm have shown optimal in vitro uptake by macropinocytosis and phagocytosis [17]. In general, pEGFP-N1 leads to the smallest NPs size and the highest *ζ*-potential in the absence of peptides and in P1 NPs compared to those corresponding to the other two plasmids, mainly pSELECT-Zeo-HSV1-tk (Table 1; Fig. 5a, b). Surface charge also plays a relevant role in NPs uptake. Cationic NPs are better internalized into the cells due to the negative charges of cell surface, while neutrally or negatively charged NPs are less efficiently internalized by the different cells [16]. This could be related to the efficiency of internalization triggered by P1 (which contains more lysine residues than P2). These analyses suggest that pEGFP-N1 provides a higher electrostatic interaction which is reflected in smaller sizes and higher *ζ*-potential values.

On the other hand, the PdI (Fig. 5c) showed that for the three plasmids NPs, the size population can be considered homogeneous in all cases (< 0.7) [18]. This suggests that the addition of the karyophilic peptides does not make a negative impact in particle agglomeration, and this will not interfere with the systems stability.

The morphology of NPs was confirmed to be spherical for both pEGFP-N1 P1 and P2 NPs and for pSELECT-Zeo-HSV1-tk NPs (Fig. 6). However, in the case of pQBI25 P2 NPs, a kind of film can be observed beneath spherical forms that apparently are NPs. This can be attributed to the fact that for SEM analysis, it is necessary to dry the sample, leading to NPs aggregation [19]. Size and PdI values determined by DLS confirm this hypothesis, showing an expected behavior for functional NPs. Moreover, pH modification could minimize the agglomeration for further morphology characterization by SEM [19].

In terms of biological functionality, NPs of all three plasmids that had the karyophilic peptides were able to get into the cell within 30 min (Fig. 7), in contrast to the control NPs without NLS, which did not show nuclear internalization before 60 min. Despite NLSs are strategies to enhance the capability of polyplexes to enter the nucleus, these results prove that they also improve the interaction with the cell membrane and further endocytosis. This can be attributed to the capability of the karyophilic peptides to penetrate cell membranes through their arginine and lysine residues, which interact with the cell membrane [20]. This also suggests that some peptide residues could be exposed in the perimeter of NPs after the interaction of the complexes pDNA-peptide with CS. Thus, both karyophilic peptides are equally efficient in improving NPs internalization at the plasma membrane level.

Qualitative results of transfection assays using pEGFP-N1 and pQBI25 NPs reveal a higher fluorescence on P2 NPs samples compared to the control and P1 NPs ones (Fig. 8). In the case of pQBI25, there was a significant difference between the size of control *vs* P2 NPs. This transfection increase could be attributed to the smaller size, the karyophilic peptide action, or both. However, the enhanced fluorescence can primarily be attributed to the action of the P2, given the absence of significant differences on physicochemical properties between P2 NPs (pEGFP-N1) and the control sample. Therefore, in this case, size can be ruled out.

In contrast, the decrease in the green fluorescent protein expression in the P1 NPs sample could be attributed to the significantly larger size of P1 NPs compared to the control and P2 NPs. This could interfere with endocytosis as described above.

The internalization and in vitro transfection assays suggest that the green fluorescent protein expression is higher with P2 NPs, although an apparently faster internalization is carried out with P1 NPs. According to our previous work, CS NPs assembled by complex coacervation can enter cells [5]. This strongly depends on the positive charges involved in the interaction with the cell membrane. Furthermore, karyophilic peptides allow for a higher interaction and confer the capability to penetrate the membrane [20] and to enter the cell, reaching the nucleus through the classical pathway.

The suggested mechanism behind the improvement in the biological functionality of NPs mainly given by P2 karyophilic peptide is as follows: (1) Internalization through non-specific (electrostatic) and specific (through arginine and lysine) interactions of peptides with the membrane [20, 21]; (2) Once in the cytoplasm, early endosomes fuse with hydrolytic vesicles of the Golgi apparatus to form an endolysosome; (3) In the late endosome, the extra positive charges given by the peptides facilitate NPs scape due to the proton sponge effect [22]; (4) In the cytoplasm, CS dissociates from pDNA-peptide complexes and they are captured and presented to the nuclear pore complexes by dynamic interactions with cytoskeleton proteins [23]; (5) The pDNA-peptide complex enters the nucleus through importin α and β following the classical pathway of nucleus entry [6–8]; and (6) Once in the nucleus, the delivered reporter or therapeutic gene is transcribed.

On the other hand, some limitations must be considered. Although these results indicate that the addition of karyophilic peptides with specific characteristics leads to an optimization of the biological functionality of NPs, this must be confirmed in vivo. Additionally, this functionality may vary depending on the physiology of each cell line.

## Conclusion

This report presents, for the first time, the effect of karyophilic peptide sequence on the physical and biological characteristics of low MW CS-based NPs using three different plasmids and the coacervation method assembly. The incorporation of peptides with NLSs to CS-based NPs formulated with different plasmids can significantly improve their physical characteristics, as well as endocytosis, nuclear internalization, and biological functionality. However, it is important to consider the characteristics of these peptides and determine of the optimal molar ratios based on the plasmid. Thus, this strategy is efficient for the optimization of nonviral vectors for gene delivery. Finally, P1 leads to an early internalization by HeLa cells, whereas P2 results in higher transfection efficiency for these reported systems.

## Data Availability

The data sets used and/or analyzed during the current study are available from the corresponding author upon reasonable request.
